# Principles of Nanoparticle Delivery to Solid Tumors

**DOI:** 10.34133/bmef.0016

**Published:** 2023-03-31

**Authors:** Warren C. W. Chan

**Affiliations:** Institute of Biomedical Engineering, University of Toronto, Toronto, ON M5S 3G9, Canada.; Terrence Donnelly Center for Cellular & Biomolecular Research, University of Toronto, Toronto, ON M5 3E1, Canada.; Department of Chemistry, University of Toronto, Toronto, ON M5S 3H6, Canada.

## Abstract

The effective treatment of patients with cancer hinges on the delivery of therapeutics to a tumor site. Nanoparticles provide an essential transport system. We present 5 principles to consider when designing nanoparticles for cancer targeting: (a) Nanoparticles acquire biological identity in vivo, (b) organs compete for nanoparticles in circulation, (c) nanoparticles must enter solid tumors to target tumor components, (d) nanoparticles must navigate the tumor microenvironment for cellular or organelle targeting, and (e) size, shape, surface chemistry, and other physicochemical properties of nanoparticles influence their transport process to the target. This review article describes these principles and their application for engineering nanoparticle delivery systems to carry therapeutics to tumors or other disease targets.

## Introduction

Advancements in engineering therapies for cancer treatment have accelerated in the past 20 years. These therapies include nucleic acids, genome editors, antibodies and proteins, and small-molecule inhibitors. They can degrade in the body, become trapped in healthy tissues, or be excreted. This results in fewer therapeutic agents targeting the tumor, leading to low therapeutic doses at the target site. Therapeutic efficacy depends on the dose of the therapeutic agent in the targeted area. The focus has shifted to designing delivery systems that efficiently transport the therapeutic agents to the target tumor tissues and cells. An example is the recent attempt to develop nanoparticle-based delivery systems to take genome editors to the cell nucleus. Researchers designed these systems to evade the liver, cellular organelles, tumor microenvironment, and other barriers [1–3].

Nanoparticles have emerged as the leading delivery technology for transporting therapeutic agents to target cells because (a) they are small enough to be transported throughout the body, (b) they can be engineered to carry different types of therapeutic agents, (c) their surface can be chemically modified with ligands to recognize cellular receptors, and (d) they can be mass-manufactured with high reproducibility for a specific size, shape, chemical composition, and surface property. Researchers have demonstrated the use of nanoparticles to carry different payloads for cancer therapy in small animal models [4–6]. However, the translation of nanotechnology for targeting and treating cancer in human patients has been limited. Less than 20 nanoparticle formulations have advanced for treating human patients with cancer. The health agencies approved most nanoparticle formulations based on the altered toxicological profile of the therapeutic agent rather than their enhanced therapeutic efficacy [7,8].

The poor delivery efficiency is one of the problems for translating nanomedicines. Wilhelm et al. [9] showed that less than 0.7% of administered nanoparticles are delivered to solid tumors. Most nanoparticles become trapped in nontumor organs, resulting in an insufficient drug dose delivered to the targeted site to elicit an effective response. In preclinical animal models, one can compensate for the low delivery efficiency by administering more nanoparticles to induce a therapeutic effect. The same strategy might not be applicable to human patients because it may lead to adverse side effects.

Solving the delivery challenge is an important objective in the 21st century. There is a need to focus on developing rationales, strategies, or blueprints to guide the engineering of delivery vehicles. This development requires a complete understanding and mapping of the physicochemical interactions of the nanoparticles with tissues, cells, and biomolecules after administration. The results of these studies will lead to correlative relationships between the particle properties and their interaction with biology. These correlations will define the parameters to build the nanoparticles for in vivo delivery and targeting applications. This research area is called the nano–bio interaction. This review article describes 5 general principles learned of the nano–bio interactions during the journey of the nanoparticles from administration to their arrival at solid tumors thus far. The nanoparticles will bind to biomolecules in serum to change their chemical identity and interaction with nontumor organs. If they escape, then they will continue to transport through the vasculature and enter the tumor microenvironment through blood vessels and navigate through the tumor and cellular components to reach cancer cells or organelles. Last, the physicochemical properties of the nanoparticles, such as the size, shape, and surface chemistry, influence their journey. We will be able to provide more precise details of the physicochemical interactions that govern the transport of nanoparticles in vivo with further investigations. These studies will refine these principles.

## First Principle: Nanoparticles Will Acquire a Biological Identity

The first principle of nanoparticle delivery to tumors posits that nanoparticles will interact with biomolecules, including proteins, lipids, and ions, after in vivo administration. Nanoparticles may also stick to cells in circulation. The most well-studied nanoparticle interactions are with serum proteins. A protein corona is called the layer of serum proteins that coats the nanoparticle surface [10,11]. We also use the term biological identity to describe the overall physicochemical properties of nanoparticles in biological media because there may be additional changes in the nanoparticle properties than the surface-adsorbed proteins [12]. For example, the nanoparticle may agglomerate into multimers. A cell sees the protein corona and unique particle morphology.

Nanoparticles instantaneously adsorb serum proteins after intravenous administration to form a protein corona. The corona composition can change when they travel in the bloodstream until the proteins reach an exchange equilibrium on the nanoparticle surface [13,14]. Researchers theorize that the bound serum proteins mediate the nanoparticles’ cellular interactions as they travel through the bloodstream. Chithrani et al. [15] provided the first reported impact of serum protein adsorption on nanoparticles and cellular uptake. They proposed that serum proteins would affect the size-dependent uptake of gold nanoparticles in cultured HeLa cells. They showed the serum protein presence on the nanoparticles by showing a shift in the agarose gel bands of nanoparticles incubated with and without serum. Cedervall et al. [16,17] used polyacrylamide gel electrophoresis to show that polystyrene nanoparticles bound many different serum proteins. They coined the term protein corona to describe the adsorbed proteins. They further described the corona as being hard or soft, referring to the strength of the nanoparticle–protein interaction. A hard corona usually has strong interactions between the nanoparticle and serum proteins, while a soft corona refers to nanoparticles that can readily adsorb and desorb from the surface. A soft corona can be called a transient protein corona.

**Fig. 1.**
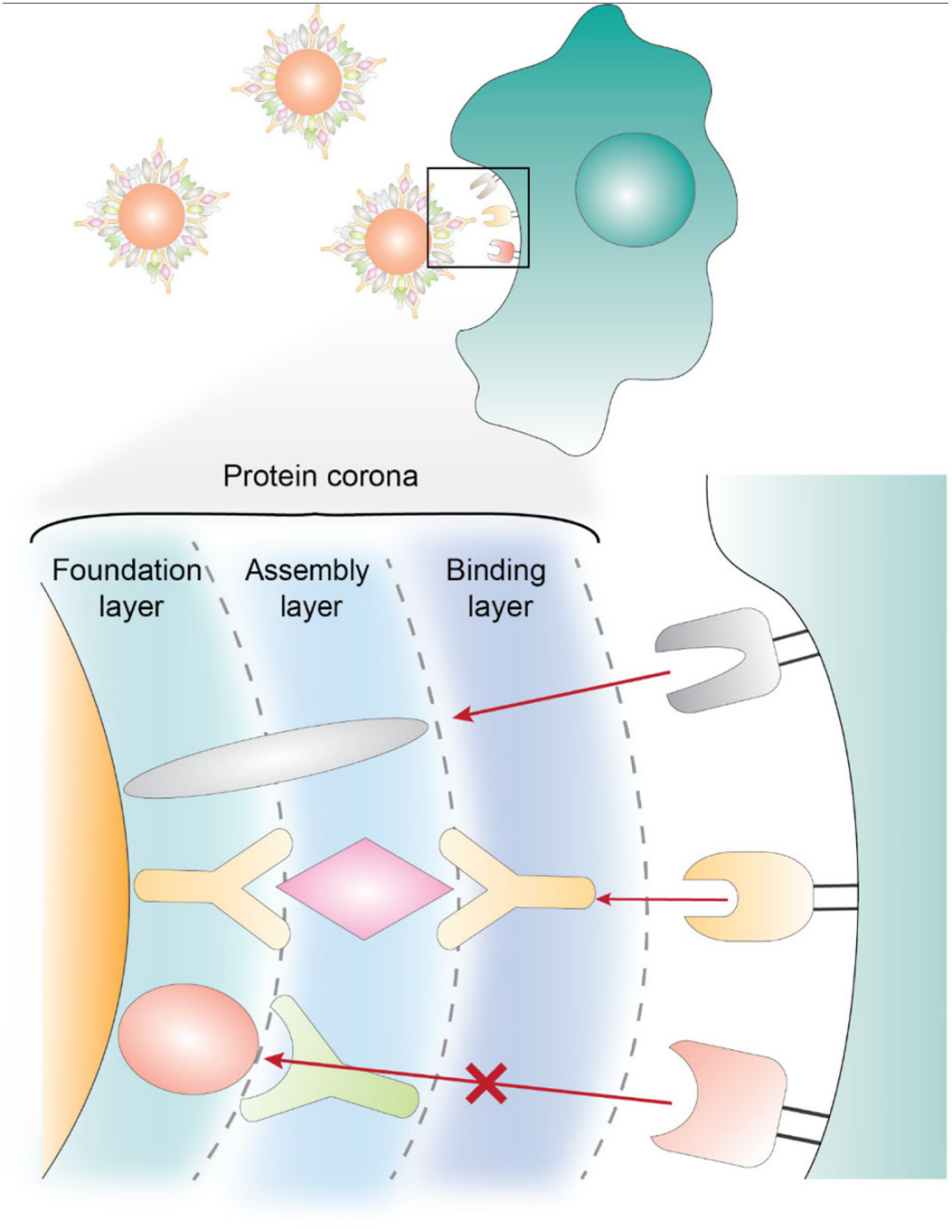
Nanoparticle biological identity. Nanoparticles become coated with proteins and other molecules from serum after intravenous administration. These proteins are organized on the nanoparticle surface, and proteins in the outermost layer can interact with cellular receptors. The surface can also contain weakly bound proteins via noncovalent and nonspecific interactions, making the protein composition dynamic. The figure is adapted from Zhang et al. [19]. Reprinted with permission from the American Chemical Society.

In 2011, Walkey et al. [11] and Tenzer et al. [18] presented the first quantitative mass spectrometry analysis of the nanoparticle protein corona. These methods enabled the researchers to identify the functional consequences of the corona. Walkey et al. [11] presented the first correlation between the nanoparticle protein corona and size-dependent macrophage uptake. Tenzer et al. [18] grouped the corona proteins by pathobiological responses. In 2020, Zhang et al. [19] showed the arrangement of corona proteins on the nanoparticle surface and how the corona architecture affected their binding to cell and tissue targets. A hard corona can potentially contain a foundation, assembly, and binding layer (see Fig. 1). The foundation layer is the layer of proteins directly bound to the nanoparticle surface, which can bind to complementary proteins (such as an antibody–antigen interaction). The binding layer has proteins that bind to cells and tissue receptors. The assembly layer contains proteins that join multiple proteins on the nanoparticle surface. The foundational layer can be the binding layer if it has proteins that bind to the cellular target. Once a hard corona forms, serum proteins can still bind and interact with the nanoparticle surface, but the binding affinity is weaker. The binding affinity of hard corona is predicted to be nanomolar, while that of soft corona is micromolar.

The role of the biological identity in mediating the nanoparticle transport properties in vivo is not fully understood. Research efforts have shifted to probing the receptors responsible for the corona’s impact on nanoparticle cell uptake and binding during circulation. Using chemical inhibitors, Lara et al. [20] started to identify the cell receptors responsible for binding the proteins on the corona. Ngo et al. [21] developed a genome screening, STRING (Search Tool for the Retrieval of Interacting Genes), and mass spectrometry technique to identify the corona ligand–cell receptor pairing in determining nanoparticle transport in vivo. They found that apolipoprotein B and low-density lipoprotein receptors are responsible for some of the nonspecific uptake of nanoparticles in healthy tissues in vivo. The availability of those techniques will enable researchers to begin defining the specific protein–protein interactions that mediate the in vivo transport of nanoparticles.

## Second Principle: Organs Will Compete with Tumors for Nanoparticles in Circulation

The second principle of nanoparticle tumor delivery posits that many organs contain cells that will compete for circulating nanoparticles. During circulation, the nanoparticles will encounter many different cell types. Approximately 30% to 99% of circulating nanoparticles may be sequestered in the organs of the reticuloendothelial system (RES). The more nanoparticles that become sequestered by nontumor cells, the less efficient the delivery process will be. A strategy to enhance nanoparticle tumor delivery is to either reduce the uptake by nondiseased tissues and cells or improve nanoparticle transport and retention into the tumor.

**Fig. 2.**
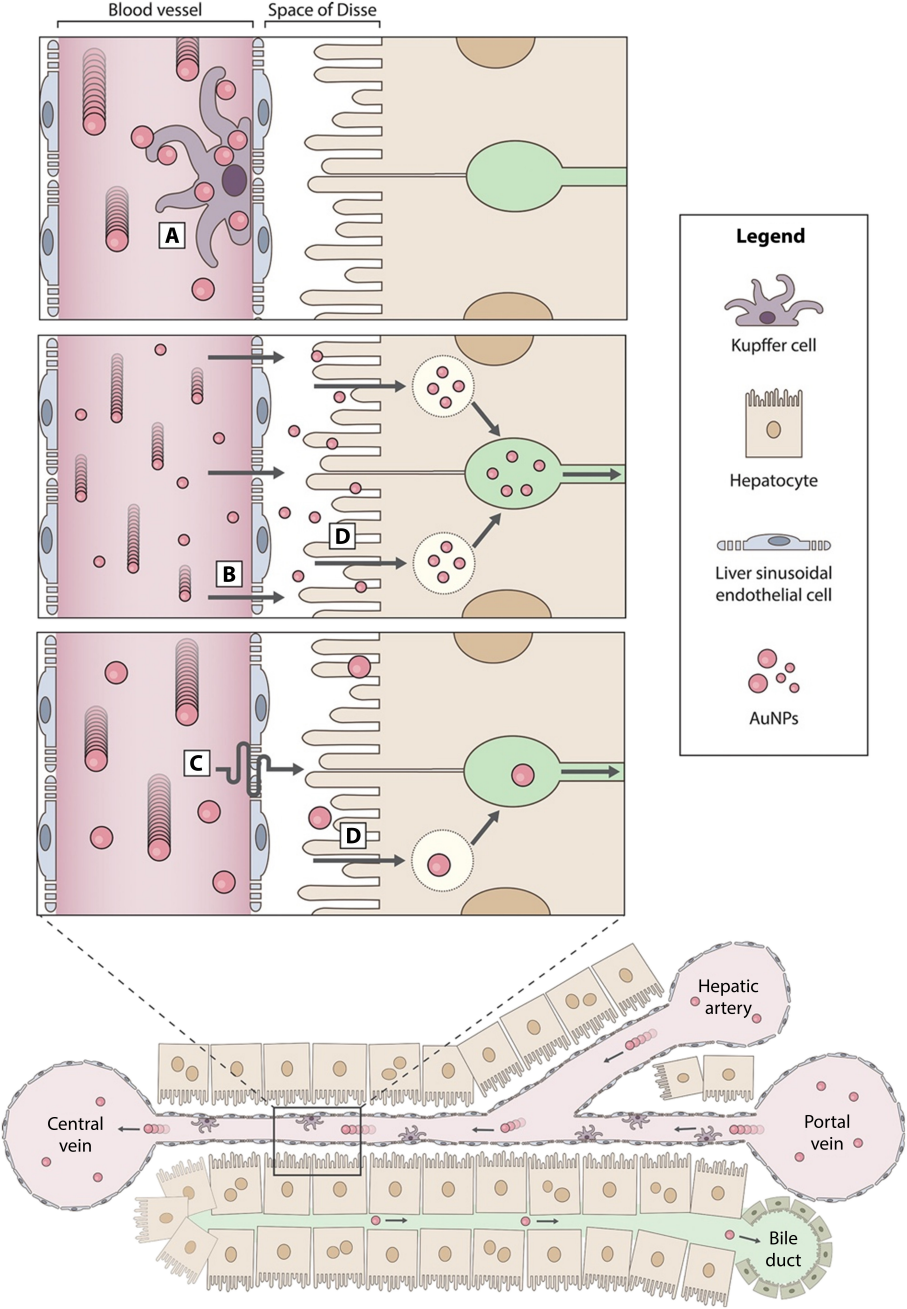
Mechanism of liver sequestration of nanoparticles. Nanoparticles enter the liver sinusoid and encounter Kupffer cells that can take them up (A). If they escape the Kupffer cells, then they can leave the liver through the central vein or interact with other cells in the liver by transporting through the liver endothelium (B and C). If they pass through the space of Disse, the body may excrete them through a fecal pathway (D). This figure is derived from Poon et al. [23]. Reprinted with permission from the American Chemical Society. AuNPs, gold nanoparticles.

The liver is the largest RES organ responsible for removing foreign particulates. As a result, this organ sequesters many nanoparticles. As nanoparticles travel into the liver through the portal vein and hepatic artery, they eventually enter the sinusoid (Fig. 2). The flow rate starts to slow in the sinusoid, increasing the chances of liver immune cells (i.e., Kupffer cells) sequestering them [22]. Kupffer cells troll the sinusoid and take up nanoparticles by a receptor or nonreceptor-mediated phagocytosis. Enzymes within the Kupffer cells can degrade lipid, polymer, or other organic nanoparticles but have greater difficulty degrading inorganic nanoparticles [23–26]. The nanoparticles also interact with liver sinusoid endothelial cells, travel into the space of Disse, interact with hepatocytes, or get eliminated if Kupffer cells do not take them up. The most likely exit path for nanoparticles from the liver is through the central vein. Once released in general circulation, the nanoparticles will re-enter the liver in the next pass, and more nanoparticles will be removed from circulation. As this process repeats, most nanoparticles are removed from circulation, degraded, or eliminated.

The molecular mechanism of how and why Kupffer cells interact with nanoparticles is still being investigated. A primary research focus is to identify the Kupffer cell receptors binding to the nanoparticle corona proteins. There is evidence that scavenger receptors are involved [24,27,28]. In parallel, researchers are developing strategies to prevent or reduce nanoparticle interaction with Kupffer cells. One method is to coat nanoparticles with neutral-charged polymers, such as polyethylene glycol (PEG), to reduce or deter serum proteins from binding to the nanoparticle surface [29–31]. The ability to prevent serum protein adsorption is related to the density and length of the PEG-coated onto the nanoparticle surface. The circulation lifetime of nanoparticles increases proportionally with the degree of PEGylation on the nanoparticle surface. Perrault et al. [32] determined that 2-kDa PEG-coated 100-nm gold nanoparticles had a faster half-life than 20-kDa PEG-coated 100-nm gold nanoparticles. Another approach to limiting the uptake of nanoparticles by Kupffer cells includes saturating the Kupffer cells with nanoparticles. Saturation can occur by injecting a nanoparticle dose that exceeds a threshold. The value is over 1 trillion nanoparticles for a mouse model [33]. Surpassing the dose threshold leads to a longer blood half-life, allowing more nanoparticles for tumor delivery. Human patients likely have a higher dose threshold than a mouse model.

Nanoparticles that escape the liver can also interact with immune cells in other organs, including the spleen, lymph nodes, bone marrow, and lung cells. Tavares et al. [34] showed that removing Kupffer cells resulted in the spleen taking up more gold nanoparticles. They administered clodronate liposome to mouse tumor models to deplete the Kupffer cells and imaged the fluorescently labeled nanoparticles in different tissues after 24 h. They found significantly more 50- and 200-nm gold nanoparticles in the spleen. The results suggest a compensatory filtration function in nanoparticle removal by the different RES organs when one organ malfunctions. The pathophysiology, cell system, and protein corona likely mediate these interactions as these organs compete with the tumor to take up nanoparticles, resulting in low delivery of nanoparticles to the tumor. A low availability of nanoparticles means a decreased transportation of therapeutic payload. This low dose at the targeted site may mean insufficient drug accumulation in target tumors to elicit a desired response.

## Third Principle: Nanoparticles Must Enter Solid Tumors for Effective Delivery

The third principle of nanoparticle tumor delivery posits that nanoparticles must enter the tumor for effective delivery. The total number of nanoparticles delivered to the tumor is the sum of the number of nanoparticles in the tumor blood vessel and microenvironment. The drug dose can be calculated by multiplying the number of nanoparticles in the tumor by the drug amount per nanoparticle. The nanoparticles likely need to cross the tumor blood vessel for high delivery.

**Fig. 3.**
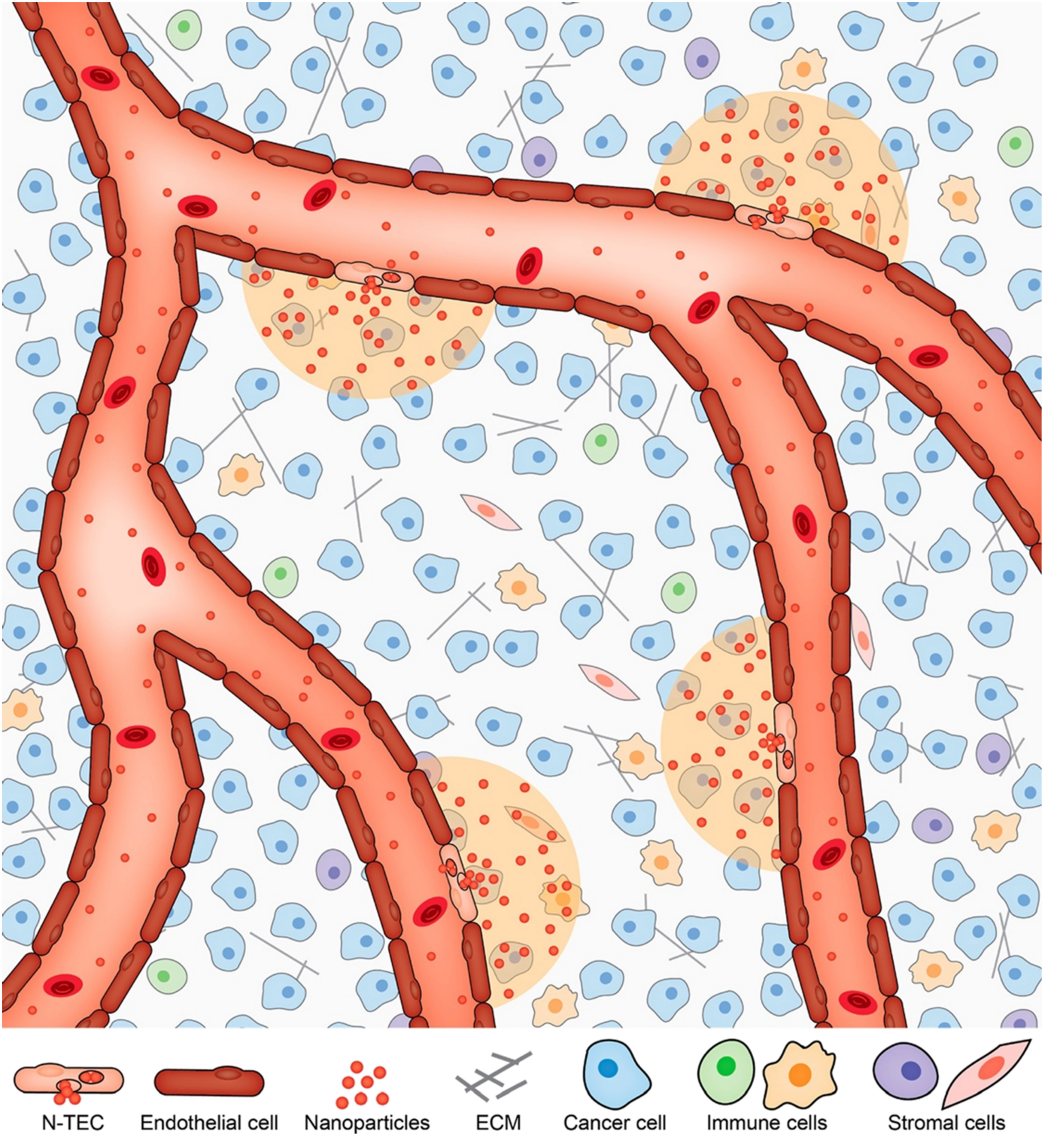
Nanoparticles entry through the tumor endothelial. Nanoparticles transport in the tumor blood vessel and can enter the tumor microenvironment. The most common mechanism of entry is through or between the endothelial cells. The site of entry affects the distance by which it must transport to reach the tumor cells. This figure is derived from Kingston et al. [43]. Reprinted with permission from the American Chemical Society. N-TECs, nanoparticle transport endothelial cells; ECM, extracellular matrix.

The nanoparticles that escape the RES organs or are not eliminated from the body have the potential to cross the tumor endothelium to the microenvironment. It was hypothesized that nanoparticles enter solid tumors through gaps between the blood vessels. When cancerous tissues undergo rapid vascularization, the vessels grow irregularly and rapidly [35]. Tight junctions do not fully form, leading to the formation of interendothelial gaps. Hobbs et al. [35] found that the size cutoff for nanoparticle transport into tumors in 7 mouse models ranged from 200 to 1,200 nm but found that the MCaIV model had an upper limit of 2,000 nm. Thus, we conclude that the gap size is conventionally smaller than 2,000 nm in mouse models and depends on the tumor type and stage. The presence and sizes of these gaps have not been thoroughly investigated in human tumors directly. Sindhwani et al. [36] did not find gaps in tissue samples from human patients with breast, ovarian, and glioblastoma cancer from electron microscopy analysis. Researchers expected that nanoparticles smaller than the gap size would diffuse through them [37–39] into the tumor microenvironment. This passive transport mechanism is central to the enhanced permeability and retention principle. The proposed mechanism led researchers to focus on engineering particles smaller than the interendothelial gaps and designing their therapeutic and imaging functions (e.g., engineering nanoparticles to diagnose and treat diseases simultaneously). However, published review and perspective articles have questioned the enhanced permeability and retention principle [40–42]. Researchers only recently presented original data to challenge this mechanism. In 2020, Sindhwani et al. [36] suggested that up to 97% of nanoparticles actively transport into solid tumors through endothelial cells. Therefore, the primary mechanism is active, not passive. Kingston et al. [43] discovered that less than 20% of the tumor endothelial cells transport nanoparticles. They named these cells nanoparticle transport endothelial cells or N-TECs (Fig. 3). They showed that the N-TEC’s locations in the tumor vessel affect the nanoparticle distribution pattern in the tumor microenvironment. N-TECs express high numbers of genes involved in endocytosis, with the highest expression being clathrin-mediated transport pathway genes. More studies are required to understand how and why N-TECs mediate transport. Lin et al. [44] recently showed that nanoparticles transport into endothelial cells in injured blood vessels through platelet factor 4 binding to receptors. Platelet factor 4 releases into the blood vessel after cellular injury and binds to the nanoparticle surface, which provides a specific ligand on nanoparticles to bind to the endothelial cell receptors. It would be interesting to determine whether the findings from Lin et al. [44] drove the nanoparticle binding and uptake into N-TECs.

There are other reported transport mechanisms for nanoparticles into the tumor microenvironment. Nanoparticles can transport through vesicular–vacuolar organelles [45,46], a channel formed by the fusion of a series of vesicles in the endothelial cells. These channels are dynamic compared to the gaps between endothelial cells. Another proposed mechanism is that nanoparticles trail behind and enter the tumor microenvironment when cells enter the tumor blood vessel. These cells enter through the tumor blood vessel, creating a transient gap for nanoparticle transport into the tumor. Naumenko et al. [47] showed that neutrophils squeezed through the tumor endothelium, allowing the nanoparticles to leak into the microenvironment. Other transport mechanisms are likely to exist. Understanding and manipulating the nanoparticle transport process through the tumor blood vessels are critical to increasing nanoparticle delivery.

## Fourth Principle: Nanoparticles Will Interact with Many Tumor and Cellular Components

The fourth principle of nanoparticle tumor delivery posits that nanoparticles will interact with different tumor components and cells in the microenvironment, which affects their delivery to tumor cells. The tumor microenvironment is complex and contains many different cell types, including macrophages, fibroblasts, cancer cells, and neutrophils. Most tumors have an extracellular matrix supporting the blood vessel and an interior necrotic region with many dead cells. The tumor microenvironment can have unique interstitial pressures that affect nanoparticle movements. Nanoparticles must navigate this environment to be delivered to cancer cells within the tumor.

**Fig. 4.**
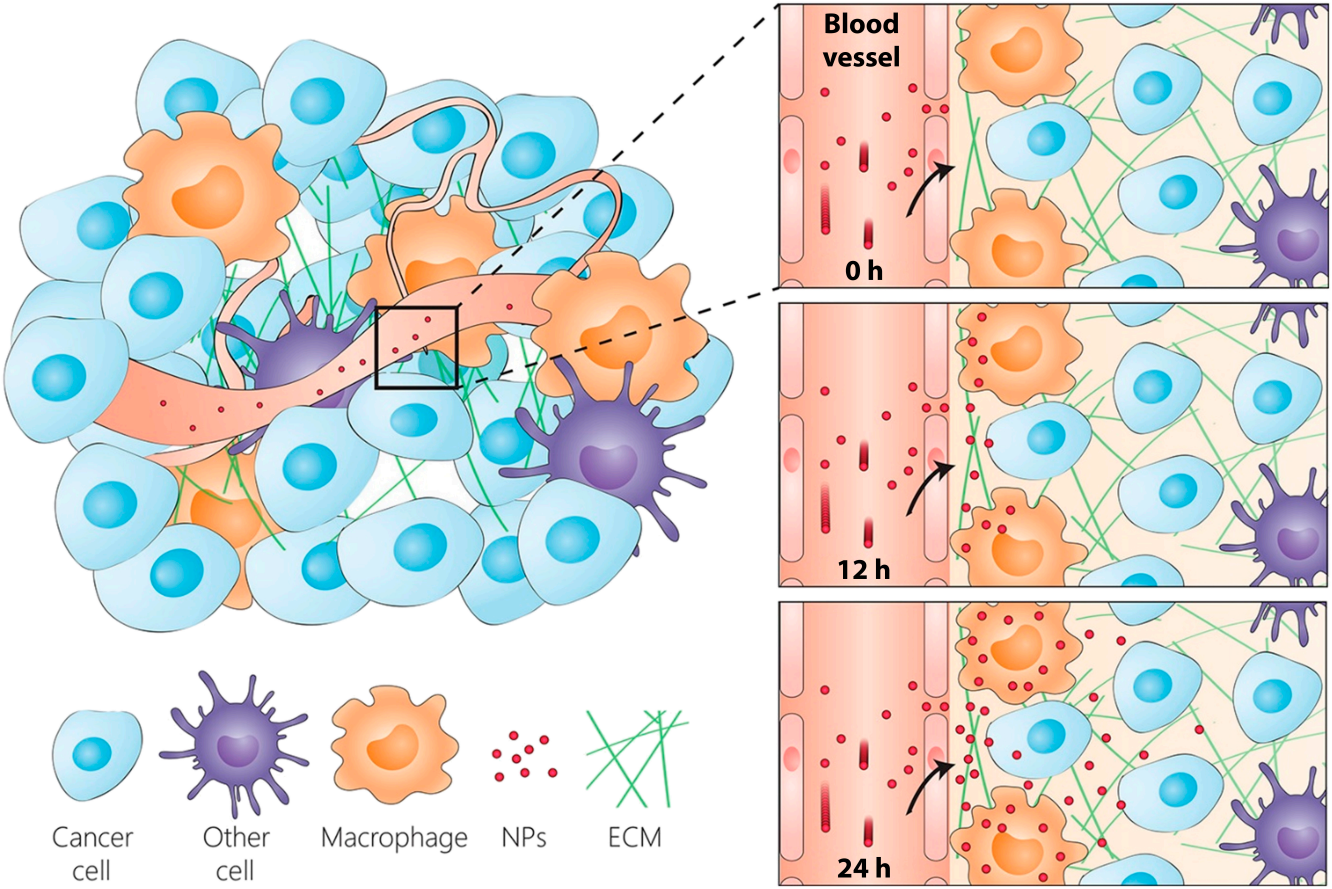
Nanoparticle interaction with cellular compartments. Upon entry into the tumor microenvironment, the nanoparticles will interact with noncancer cells, extracellular matrix (ECM), and other components. The other components will affect the number of nanoparticles delivered to the cancer cells. This figure is derived from Dai et al. [51]. Reprinted with permission from the American Chemical Society.

Specific cellular delivery of nanoparticles in the tumor depends on their ability to evade the tumor’s extracellular matrix and nontarget cells before reaching the targeted cancer cells (see Fig. 4). Surrounding the tumor endothelium is a basement membrane. This membrane contains an extracellular matrix that can trap nanoparticles and inhibit their ability to transport deep into the tumor microenvironment. Nanoparticles that are trapped in the membrane can be transported into the tumor by tumor-associated macrophages (TAMs). These cells migrate toward membrane regions with high numbers of nanoparticles after crossing the tumor vessel [48]. The TAMs take up nanoparticles and transport them deep into the tumor microenvironment. The migration of TAMs to the tumor site stops once most or all the nanoparticles have been taken up. Particle sizes appear to determine whether the nanoparticle diffuses into the tumor microenvironment or is taken up by TAMs. Smaller nanoparticles (<30 nm) are more likely to diffuse into the microenvironment. Larger nanoparticles are more prone to be taken up by the TAMs. Miller et al. [49,50] and Dai et al. [51] showed that the TAMs take up many nanoparticles after crossing the tumor blood vessel. Other tumor cells, such as cancer-associated fibroblasts, can also interact with nanoparticles in the microenvironment [52]. The tumor may contain matrices throughout the tissue, and the nanoparticles must navigate through it.

Cancer cells have been the primary target for many nanoparticle-based therapies. Researchers coat the nanoparticles with cancer cell targeting agents such as Herceptin and folic acid [53–56]. The nanoparticles face many biological headwinds in reaching the target cancer cells. Dai et al. [51] showed that less than 0.0014% of administered nanoparticles would bind cancer cells in the SKOV-3 cancer mouse model. The low cell delivery efficiency results from the busy tumor microenvironment in which many different cells and structures compete with or sequester the nanoparticles. This competition or sequestration leaves fewer nanoparticles interacting with receptors. The percentage of nanoparticles delivered to the organelles, such as the mitochondria and nucleus, is likely even less because the nanoparticles must overcome many additional cellular barriers (e.g., escaping endosomes). Significant efforts are underway to develop new nanoparticle design strategies to overcome these barriers.

## Fifth Principle: Nanoparticle Physicochemical Properties Will Influence the Delivery Process

The fifth principle of nanoparticle tumor delivery posits that the physicochemical properties of nanoparticles affect their delivery to the target site. They include the nanoparticle size, shape, surface chemistry, and other physicochemical properties. They determine the nanoparticle tumor entry rate, residence time, and penetration depth, and interactions with RES organs. There is an effort to organize the results from these nano–bio interaction studies into a searchable database where machine learning tools can determine the optimal nanoparticle formulations for targeting and delivery to specific tumors [14,57–60]. We are currently at the data-generation stage. Eventually, the database will provide information about the optimal nanoparticle properties for tumor delivery and targeting applications.

I provide examples of the impact of the physicochemical nanoparticle parameters on nanoparticle tumor delivery. Sixty-nanometer PEGylated-gold nanoparticles have 25 and 3 times more tumor accumulation than 20- and 100-nm gold nanoparticles, respectively [32]. Geng et al. [61] showed rod-shaped fibers have a longer half-life and slower macrophage uptake than spherical particles of the same chemistry. These nanofibers extend in flow, allowing them to move through the tumor matrix. Such rod-shaped particles carrying the chemotherapeutic drug paclitaxel shrunk tumors twice as much as an injection of paclitaxel. Decuzzi et al. [62] showed that the liver took up micrometer-sized cylindrical particles significantly more than spherical, hemispherical, and disc-shaped structures. They have a “larger rotational inertia and surface of adhesion, which facilitate their interaction with the vessel walls” [62]. Choi and co-workers [63] showed that semiconductor nanocrystals below 6.0 nm were excreted, while nonrenal organs can trap larger sizes. Nanoparticle size also influences their short- and long-term liver uptake. Still, each researcher used differing particle designs to illustrate these principles. There is a need to develop a universal database system for data input and mining.

At the cellular level, the physicochemical properties of nanoparticles can affect cellular response and biology. In vitro cell studies strongly indicate nanoparticle size and shape dictate many cellular responses. Studies showed the uptake and removal into and out of a cell depend upon the nanoparticle’s geometry [64–67]. HeLa cells appeared to take up ~50-nm gold nanoparticles at the fastest rate and highest concentration in culture compared to other sizes. However, removing the gold nanoparticles from the cell had a linear relationship, where small nanoparticles exited cells faster [68]. Ho et al. [69] showed that the mechanism of gold nanoparticle exiting is due to exosome transport. This transport process may be size dependent, as the exosomes have defined sizes to encapsulate the gold nanoparticles. The next important step is to model these nanoparticle–cell processes to elucidate and predict the interactions based on the particle’s physicochemical properties. One study demonstrated that nanoparticle sizes correlated with the internalization behavior of the surface receptor and the subsequent intracellular signaling events and toxicity of Herceptin [70]. Although most studies were conducted in an in vitro culture model, they definitively demonstrated that the size of the nanoparticle is essential in dictating cellular activities, function, and response. A thorough understanding of how particle design impact’s cellular function and behavior can lead to new therapeutics. It is crucial to identify designs that do not perturb biological systems too much for delivery applications—as such, effects could lead to toxicity.

A strong focus remains on identifying the optimal physicochemical nanoparticle parameters for cellular delivery. Many gene therapies, small-molecule inhibitors, and other therapeutic systems require nanoparticles to deliver to target cells and organelles successfully. Identifying the optimal design can be achieved by (a) systematically investigating the role of these parameters in cellular delivery and intracellular targeting, (b) determining the impact of nanoparticle design on cellular function and signaling, (c) quantifying the delivery efficiency to the final target, and (d) using artificial intelligence to discern transport patterns. One can envision that one day, in the not-too-distant future, a researcher/clinician will be able to input a target of interest into a database, and a piece of software will output the optimal nanoparticle design to deliver a therapeutic payload to the target.

## Summary

In the past 30 years, we have witnessed significant advancements in the development of nanoparticle delivery systems. We developed methods to prepare inorganic nanoparticles (e.g., gold nanoparticles and silver nanoparticles) and organic nanoparticles (e.g., liposomes, exosomes, and viruses). We created new chemistry to coat them with various molecules (e.g., polymers, antibodies, and peptides). We have engineered the nanoparticles to mimic biology, such as coating nanoparticles with cell membranes. After the development of methods for nanoparticle synthesis, researchers focused their efforts on using these nanoparticles to deliver cancer therapeutics. With the limited translation of nanoparticle formulations, researchers started to refocus their energy on probing the nanoparticle delivery process. This effort has led to a better understanding of nanoparticle behavior in the transport process. Fundamentally, all nanoparticle designs face barriers to reaching the target tissues. This article distilled these results into the 5 principles of nanoparticle delivery to cancer cells.

While the broad framework of these principles has taken shape, the specific details that underpin it require further investigation. Mathematical equations are starting to be formulated to describe the nanoparticle delivery process [71]. These equations may become part of a computational approach to helping predict and identify the optimal formulations for cancer delivery. The results will together shape the rules for engineering nanoparticle delivery systems. Although tumors were the primary disease models in these studies, the principles learned from nanoparticle targeting to solid tumors can be adapted for cardiovascular, cystic fibrosis, and other diseases. Moving forward, we must continue to expand our understanding of the interactions between nanoparticles and biological systems. The results will guide and refine nanoparticle design principles for in vivo applications for cancer and beyond.
